# A broad-spectrum bactericidal lipopeptide with anti-biofilm properties

**DOI:** 10.1038/s41598-017-02373-0

**Published:** 2017-05-19

**Authors:** Ohad Meir, Fadia Zaknoon, Uri Cogan, Amram Mor

**Affiliations:** Department of Biotechnology & Food Engineering, Technion-Israel Institute of Technology, Haifa, 32000 Israel

## Abstract

Previous studies of the oligoacyllysyl (OAK) series acyl-lysyl-lysyl-aminoacyl-lysine-amide, suggested their utility towards generating robust linear lipopeptide-like alternatives to antibiotics, although to date, none exhibited potent broad-spectrum bactericidal activity. To follow up on this premise, we produced a new analog (C_14_KKc_12_K) and investigated its properties in various media. Mechanistic studies suggest that C_14_KKc_12_K uses a non-specific membrane-disruptive mode of action for rapidly reducing viability of Gram-negative bacteria (GNB) similarly to polymyxin B (PMB), a cyclic lipopeptide used as last resort antibiotic. Indeed, C_14_KKc_12_K displayed similar affinity for lipopolysaccharides and induced cell permeabilization associated with rapid massive membrane depolarization. Unlike PMB however, C_14_KKc_12_K was also bactericidal to Gram-positive bacteria (GPB) at or near the minimal inhibitory concentration (MIC), as assessed against a multispecies panel of >50 strains, displaying MIC_50_ at 3 and 6 µM, respectively for GPB and GNB. C_14_KKc_12_K retained activity in human saliva, reducing the viability of cultivable oral microflora by >99% within two minutes of exposure, albeit at higher concentrations, which, nonetheless, were similar to the commercial gold standard, chlorhexidine. This equipotent bactericidal activity was also observed in pre-formed biofilms of *Streptococcus mutans*, a major periodontal pathogen. Such compounds therefore, may be useful for eradication of challenging poly-microbial infections.

## Introduction

Facing the global crisis of multidrug resistant bacteria^1–6^, membrane active compounds (MACs) are earning a renewed attention for their potential to control infections^7–10^ by multiple mechanisms, including by affecting critical common bacterial processes such as communication^11, 12^ and virulence^13, 14^ at sub-inhibitory concentrations. Thus, although the molecular basis for these effects are relatively ill understood, various MACs are presently gaining interest for their potential to address antibiotic resistance challenges and promise to overcome infections while avoiding many of the known resistance mechanisms. Of particular interest are borderline-hydrophobic MACs which, at low micromolar concentrations instigate mild/transient membrane damages^15–17^, including partial loss of the transmembrane potential, believed to bare critical consequences on efflux function^18^ and expression of antibiotic resistance factors^19^. While borderline-hydrophobic MACs might exert a bacteriostatic mode of action at higher doses, outright-hydrophobic MACs tend to disrupt biological membranes structures abruptly^20–22^, which often culminates in a rapid bactericidal outcome, at or near the minimal inhibitory concentration (MIC)^23, 24^.

Many of the known antimicrobial peptides (AMPs) exert antibacterial activities over Gram-positive bacteria (GPB) and/or Gram-negative bacteria (GNB) through their MAC properties. Some, manage to breach the cytoplasmic membrane (CM) permeability barrier^20, 25, 26^ while others selectively increase the outer membrane (OM) permeability in GNB by perturbing the organization and function of the lipopolysaccharides (LPS) layer^23, 27–29^. Thus, owing to its hydrophilic attributes, the OM is often responsible for the low sensitivity of GNB to hydrophobic antimicrobials (that can be highly active on GPB), thereby further challenging the generation of broad-spectrum antibacterial compounds, particularly needed in poly-microbial infections, for instance.

A variety of strategies were proposed to generate chemical mimics of AMPs to alleviate complications associated with peptide drugs^30, 31^. AMP-mimics may also be beneficial in helping to improve understanding the complex molecular basis for AMPs function(s) owing to their relative molecular/structural simplicity. Namely, while charge and hydrophobicity represent most critical factors influencing AMP properties, it is often challenging to identify the respective optimal proportions for controlling potency or selectivity. For instance, excess hydrophobicity might promote self-assembly in aqueous solutions and consequently might reduce potency^32, 33^. Charge considerations are similarly intricate, namely due to the fact that the relative proportion of anionic phospholipids in bacterial membranes can reach 20–30% in GNB and up to nearly 100% in GPB^34, 35^. In this respect, the peptidomimetic approach using oligo-acyl-lysines (OAKs) seems particularly suitable for engineering high affinity antibacterial MACs^22, 36–38^ since OAKs composition consists exclusively of hydrophobic linear acyls and cationic lysine residues^37–39^, where the inherently simple and incremental nature of designed analogs provides a systematic tool for dissecting the relative importance of charge and/or hydrophobicity. In fact, OAK designs that concentrated on miniaturized sequences of the general formula: *Acyl-lysyl-lysyl-aminoacyl-lysine-amide* suggest its capacity to generate distinct MAC versions with rational antimicrobial behaviors. For instance, the inactive tetrapeptide H-KKc_12_K-NH_2_ became active upon conjugation of N-terminal acyls, gradually revealing increasing activity with increased acyl length. Thus, C_8_KKc_12_K or C_10_KKc_12_K remained essentially inactive^40^ unlike more hydrophobic analogs^38, 41^.

**Figure 1:**
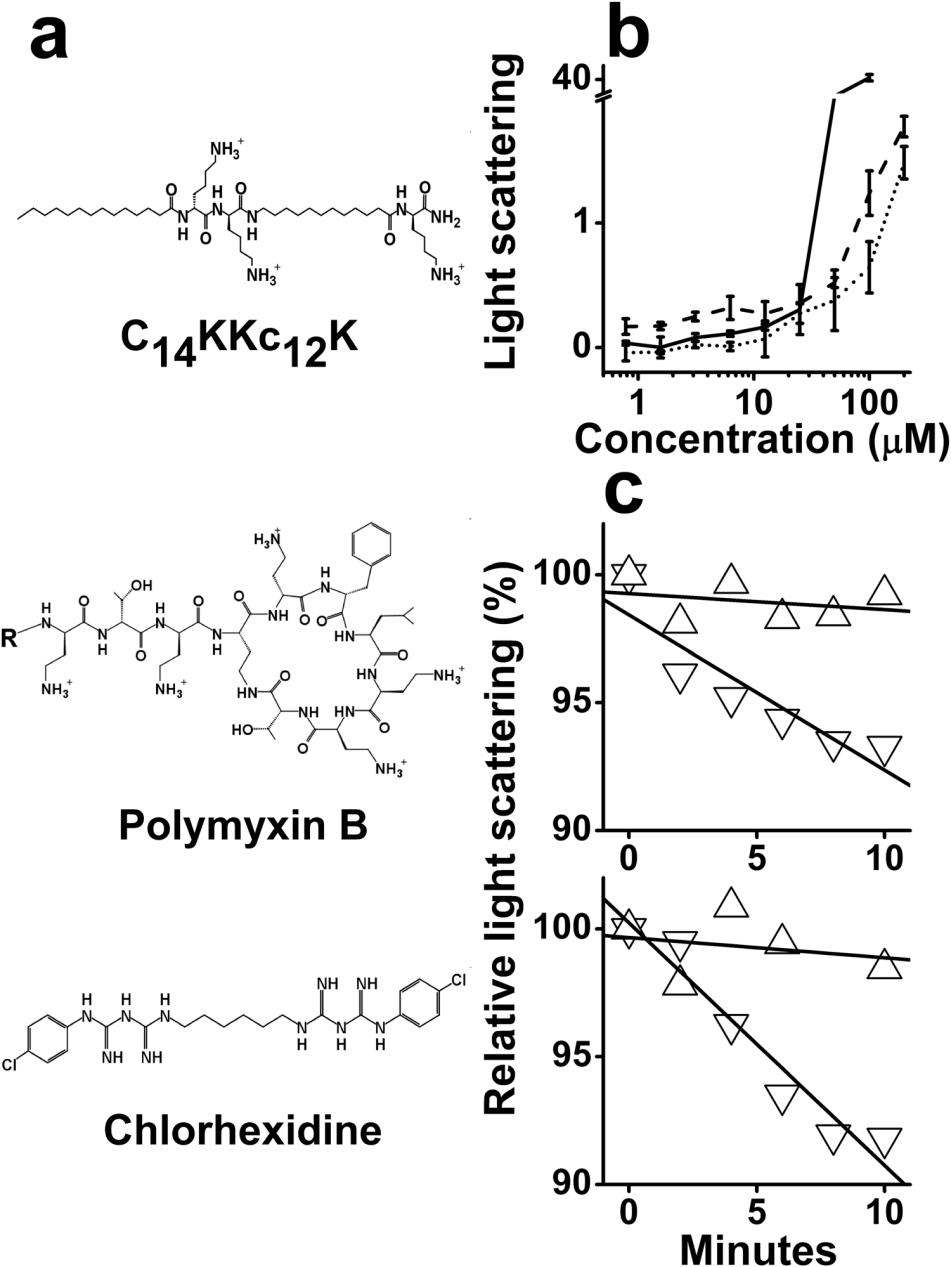
Molecular structure and organization in aqueous solution. (**a**) Structure of C_14_KKc_12_K (top), PMB (middle; R = 6-methyloctanoyl) and CHX (bottom). (**b**) Two fold dilutions of three OAKs were prepared in PBS and light scattering was measured after two hours incubation. Symbols: dotted line, C_12_KKc_12_K; solid line, C_14_KKc_12_K; dashed line, C_16_KKC_12_K. Results are from at least two independent experiments. Error bars represent the standard deviation. (**c**) Representative light scattering experiments showing the signal evolution immediately after adding 10^5^ CFU/ml (top, *S. aureus*; bottom, *E. coli*), using the conditions described in panel (**b**) (OAK at 200 µM). Symbols: triangles, C_12_KKc_12_K; inverted triangles, C_14_KKc_12_K. The vast majority of data points standard deviations varied by <2%.

Here, we set out to verify the implied linear relationship (between potency and hydrophobicity) of this C-terminally amidated series by producing a previously untested intermediate analog, C_14_KKc_12_K (molecular structure depicted in Fig. 1a) and investigating its antibacterial activity, mode of action and potential application. Ultimately, the findings are discussed in the context of a series of analogs, C_n_KKc_12_K (n = 8, 10, 12, 14 and 16).

## Results

As part of its biophysical characterization, the purified synthetic lipopeptide was first subjected to light scattering measurements in order to assess its potential for self-assembly in an aqueous environment, as compared with two analogs, C_12_KKc_12_K and C_16_KKc_12_K. Expectedly, the light scattered by these analogs at low concentrations and up to ~10 µM, displayed proportional amplitudes, where C_14_KKc_12_K was intermediate between C_12_KKc_12_K (lowest) and C_16_KKc_12_K (highest) (Fig. 1b) and started to deviate from linearity at a concentration range where both C_12_KKc_12_K and C_16_KKc_12_K were previously found to aggregate^24, 41^. At ~20 µM however, C_14_KKc_12_K revealed a sharp divergence since its light scattering pattern exhibited significantly higher amplitudes, likely to reflect its capacity to form supramolecular structures of larger sizes^42^. We also assessed the aggregation tendency in a more complex medium such as the supernatant of centrifuged saliva and found it to display an overall similar trend to that in PBS, only starting at somewhat lower concentrations (i.e., the respective critical aggregation concentrations values were 8 ± 1 and 23 ± 3 µM). Often, such self-assembly is deleterious to antibacterial potency of AMPs and OAKs^24, 32, 33^. Though, in the case of C_12_KKc_12_K and C_16_KKc_12_K, we previously used their respective unsaturated N-terminal acyls to show that the less hydrophobic counterparts (i.e., C_12ω7_KKc_12_K and C_16ω7_KKc_12_K)^24, 43^ displayed higher potencies, even as aggregates. The proposed explanation for this discrepancy was that the unsaturated analogs formed different structures where the self-assembled lipopeptides were not as tightly bound^24, 43^, thereby gaining the ability to dis-aggregate upon interaction with bacteria and the ability to exert their antibacterial activity, unlike the saturated counterparts. Consequently, we next verified the analogs propensities to disaggregate in presence of bacteria by monitoring their light scattering amplitudes. Figure 1c illustrates the rapidly reduced light scattered by C_14_KKc_12_K, indicating its greater tendency to disaggregate in presence of bacteria. While these findings raise interesting questions (such as whether higher bacterial concentrations and/or longer incubation periods will increase the OAK disassembly), the fact that C_14_KKc_12_K demonstrated a tendency for rapid disaggregation in presence of bacteria supports the rapid activity expected in certain applications (as exemplified later in this section). Collectively therefore, the data suggest that the OAK’s tendency for self-assembly in a biological milieu at high concentrations, might not interfere with its antibacterial activity.

Next, we characterized its antibacterial activity by determining the MIC against a multispecies panel of 54 representative bacteria, including various medically relevant strains. Table 1 summarizes the MIC values obtained with 30 GPB (five species) and 24 GNB (seven species). With one exception (one of the *Acinetobacter baumannii* clinical isolates), the data reveal that C_14_KKc_12_K was active on most tested bacteria, although GPB appear generally more sensitive (MIC_50_ 3 and 6 µM, respectively). Replacing LB with cation adjusted Mueller Hinton Broth resulted in essentially similar outcome. For instance, MICs of *S. aureus* 29213 or *E. coli* 25922 were 3 and 6 µM, respectively. Also note that C_14_KKc_12_K displayed an unchanged MIC value (3 µM) on both the wild type (AG100) and its efflux deletion-mutant (AG100A), unlike its analogs^18^ (this issue will be elaborated in the Discussion section).

**Table 1:** MIC of C_14_KKc_12_K against a representative panel of bacteria.

Species (number of strains tested)	MIC^a^ range (µM)
**Gram-positive bacteria**	
*Streptococci* (9)	0.78–3.12
*Staphylococci* (10)	1.56–3.12
*Enterococci* (3)	3.12–6.25
*Bacilli* (2)	6.25
*Listeria* (6)	3.12
**Gram-negative bacteria**	
*Escherichia* (9)	3.12–6.25
*Pseudomonas* (3)	6.25–12.5
*Klebsiella* (3)	3.12–12.5
*Acinetobacter* (5)	3.12–>25
*Salmonella* (1)	3.12
*Fusobacterium* (2)	6.25
*Porphyromonas* (1)	3.12

^a^MIC was determined by the microdilution method. Values represent the average of at least 2 independent experiments performed in duplicate.

**Figure 2:**
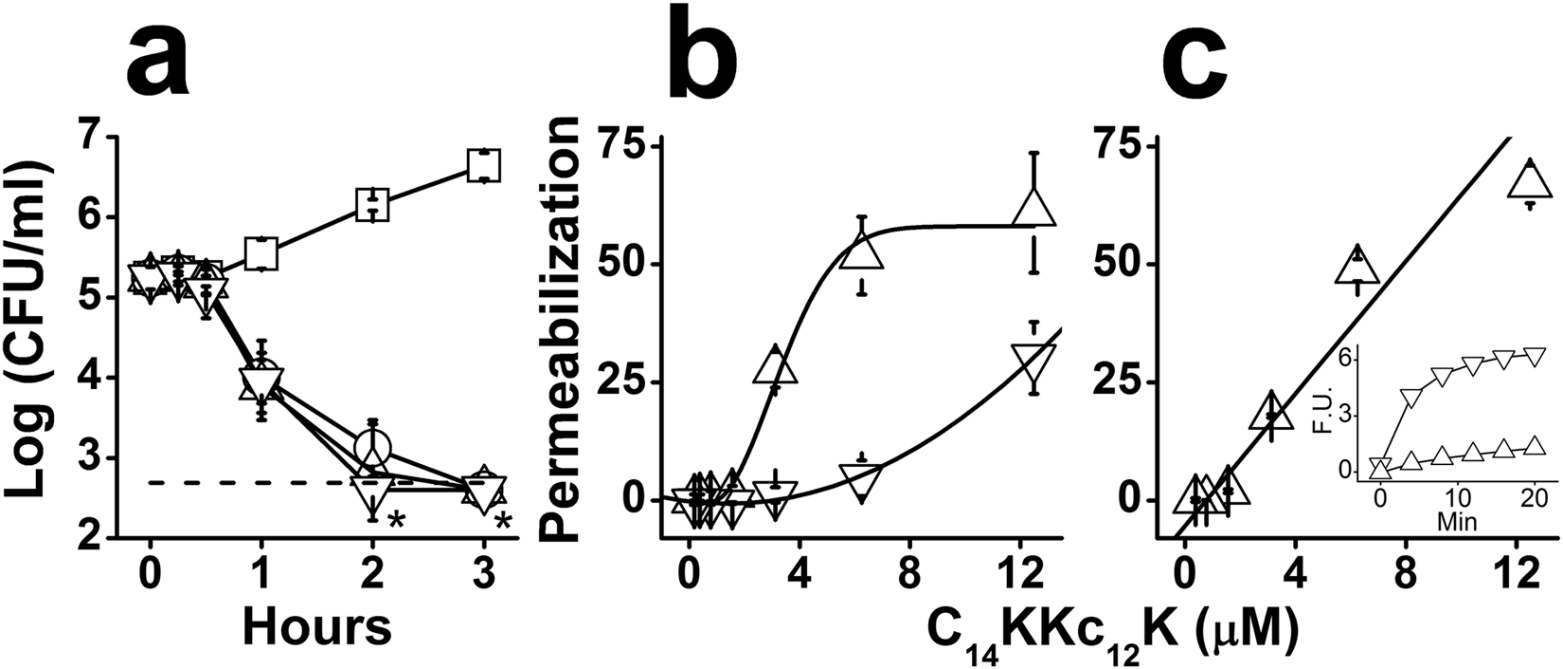
Mode of action investigated against *E. coli* ML-35p as GNB representative. (**a**) Bactericidal kinetics upon exposure to C_14_KKc_12_K 0, 1, 2 and 4 MIC multiples (squares, circles, triangles and inverted triangles, respectively); dashed line, limit of detection (500 CFU/ml); asterisks denote lack of detected CFUs. (**b**,**c**) Membrane permeabilization, expressed as percentage of the positive control dermaseptin S4 (1–15) at 6.25 µM. Symbols: triangles, outer membrane; inverted triangles, cytoplasmic membrane. (**c**) Cytoplasmic membrane permeation to ethidium bromide. The inset shows representative permeation kinetics by the OAK at the MIC (triangles) and the positive control (inverted triangles); Min, minutes; F.U., fluorescence units (excitation: 535 nm, emission: 590 nm). Results are from at least two independent experiments performed in duplicate. Error bars represent the standard deviation.

To investigate the mode of action, we used standard methodologies for MAC characterization, including assessment of membrane damages and determination of time-kill kinetics over both *Escherichia coli* and *Streptococcus mutans*, respectively representing GNB and GPB. Figure 2a shows the OAK’s ability to affect viability of *E. coli*, reflecting a rapid bactericidal mode of action (e.g., >2-log-unit reduction within 2 hours exposure at ≥MIC).

**Figure 3:**
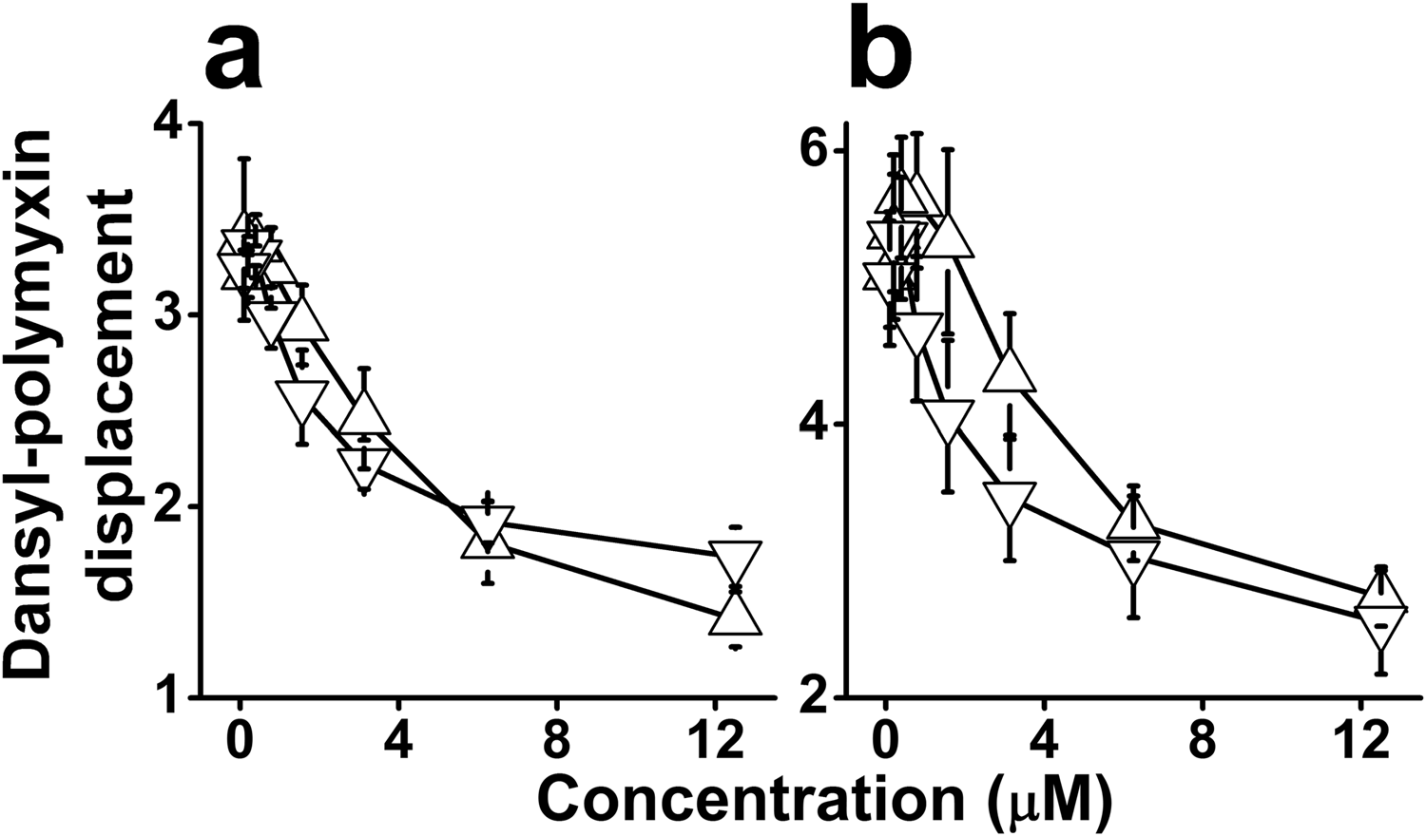
Dansyl-polymyxin binding assay. Interaction with LPS was assessed by incubation (1.5 hr) of C_14_KKc_12_K and polymyxin B with 2 μM pure monodansyl-polymyxin and 3 μg/ml LPS from *E. coli* (**a**) or *P. aeruginosa* (**b**). Symbols: triangles, C_14_KKc_12_K; inverted triangles, Polymyxin B; The Y axis represents fluorescence measurements (excitation: 340 nm, emission: 485 nm). Results are from two independent experiments performed in duplicate. Error bars represent the standard deviation.

To assess potential membrane damages, we used an assay capable of differentiating permeability changes in bacterial membranes by testing the leakage of small organic molecules. The assay employs the engineered *E. coli* strain, ML-35p, which is constitutive for cytoplasmic β-galactosidase, lacks lactose permease, and expresses a plasmid-encoded periplasmic β-lactamase^44^. The chromogenic β-galactosidase substrate ONPG is used to assess permeation of the CM, while OM permeability is assessed using nitrocefin, a chromogenic β-lactamase substrate. The data summarized in Fig. 2b suggest that both membranes were permeabilized at the MIC value (3 µM) albeit the OM appears more susceptible. To validate the CM damages at low concentrations, bacteria were subjected to another permeability assay, this time monitoring cytoplasmic access to the DNA binder, ethidium bromide (Fig. 2c). The fact that ethidium bromide accumulated in *E. coli* provides confirming evidence for the CM permeabilization at the MIC value. The inset shows two representative kinetic curves illustrating ethidium bromide’s rapid accumulation in bacterial cytoplasm. Combined, these findings support the view that bacterial death has resulted from the OAK’s capacity to abruptly disrupt both membranes. A similar mode of action was attributed to the natural bacteria-derived 11-residue cyclic lipopeptide, polymyxin B (PMB)^45^, an effect believed to stem from its high-affinity interaction with LPS. When compared, PMB and C_14_KKc_12_K exhibited a similar binding affinity to LPS originating from *E. coli* or *Pseudomonas aeruginosa*, as determined by their abilities to displace the binding of dansyl-PMB (Fig. 3). Under similar conditions^46^ the analogs C_8_, C_10_ and C_12_ exhibited a binding affinity that was lower than that of C_14_KKc_12_K but increased with increasing hydrophobicity^40^. This is further addressed below.

**Figure 4:**
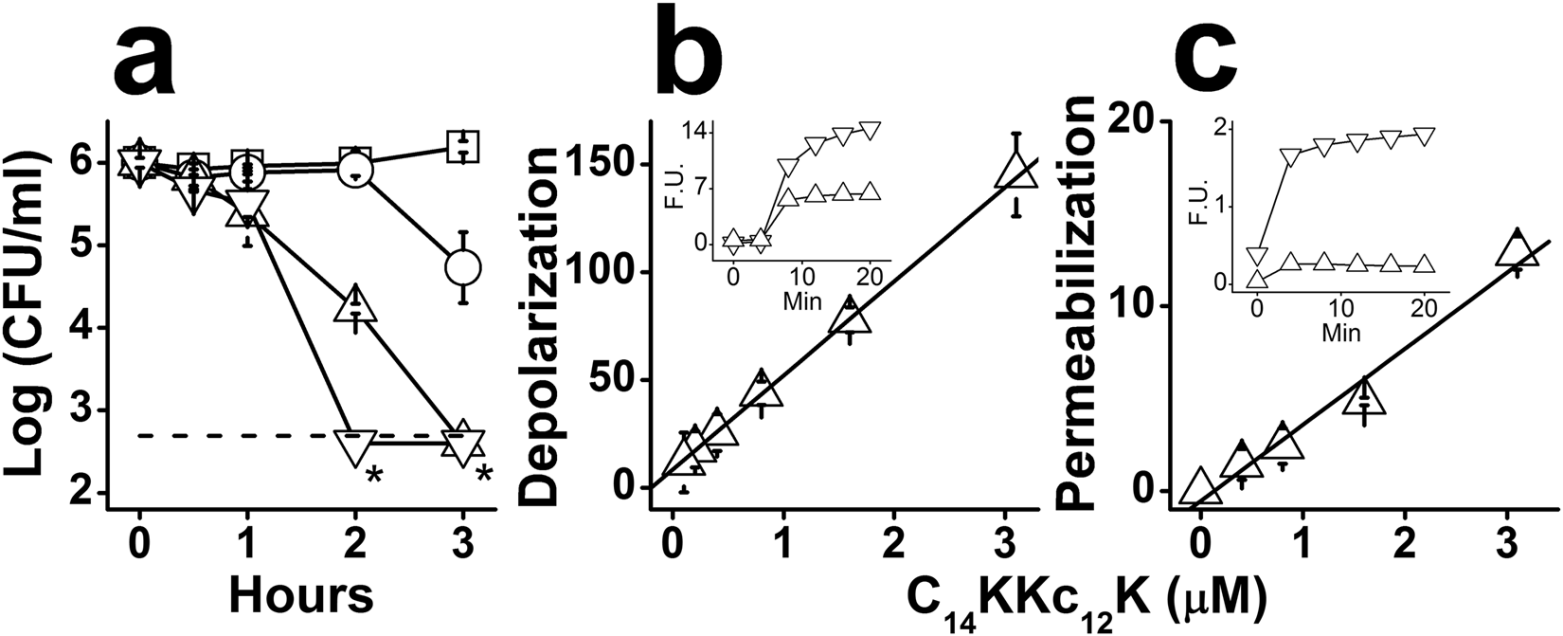
Mechanistic studies (GPB). C_14_KKc_12_K mode of action was investigated against *S. mutans* ATCC 35668 as GPB representative. (**a**) Bactericidal kinetics upon exposure to 0, 1, 2 and 4 MIC multiples (squares, circles, triangles and inverted triangles, respectively); dashed line, limit of detection (500 CFU/ml); asterisks denote lack of detected CFUs. (**b**,**c**) Membrane damages instigated by C_14_KKc_12_K, expressed as percentage of the positive control dermaseptin S4(1–15) at 6.25 µM. Membrane depolarization (**b**) assessed by displacement of DiSC_3_(5), and membrane permeation (**c**) assessed by accumulation of EtBr. The insets in (**b** and **c**) show representative depolarization and permeation kinetics, of the OAK (triangles) and the positive control (inverted triangles) at 0.78 and 6.25 µM, respectively; Min, minutes; F.U., fluorescence units (excitation: 620 nm, emission: 680 nm in panel b; excitation: 535 nm, emission: 590 nm in panel c). Results are from at least two independent experiments performed in duplicate. Error bars represent the standard deviation.

Figure 4a shows the OAK’s ability to affect viability of *S. mutans*, reflecting, again, the OAK’s rapid bactericidal mode of action at low micromolar concentrations (e.g., ~2 log units reduction within 2 hours exposure to 1.56 µM). Membrane damages were evident from the rapid and massive leakage of protons and cytoplasmic accumulation of ethidium bromide (Fig. 4b and c, respectively).

To determine the applicative potential of C_14_KKc_12_K, we next aimed to exploit apparent OAK advantageous properties such as protease stability^38, 39^ and rapid bactericidal mode of action over a broad spectrum of bacteria. Hence, we performed a preliminary assessment for OAK’s ability to affect multiple bacterial species in saliva, given that the list of susceptible strains (**Materials & Methods** section) included various bacterial species known to promote periodontal diseases^47, 48^ whose treatment with an orally active antimicrobial has been the standard clinical approach. For this purpose, we elected to use *S. mutans*, a prototypical oral pathogen. Unlike other salivary floating bacteria, *S. mutans* can adhere to the oral cavity surfaces (particularly to teeth) and promote biofilm formation, which disturbs the balanced oral microbiome by pH reduction, culminating in dental complications such as caries and periodontal inflammations^49–51^.

**Figure 5:**
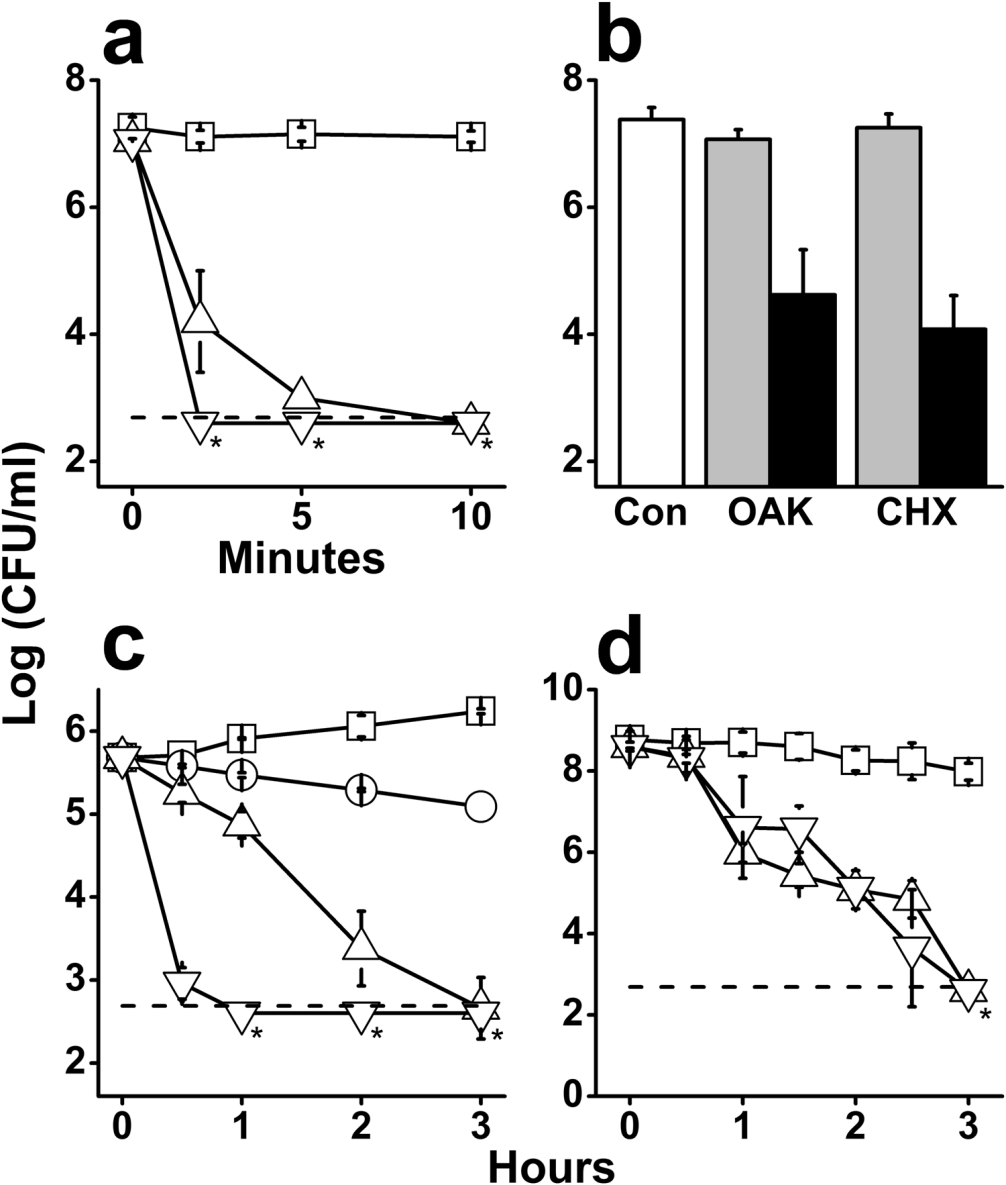
Potential oral application of C_14_KKc_12_K. (**a**) Bactericidal kinetics of C_14_KKc_12_K against *S. mutans* ATCC 35668 in the supernatant of centrifuged saliva. Symbols: squares, untreated control; triangles, C_14_KKc_12_K at 0.1 mM; inverted triangles, C_14_KKc_12_K at 1 mM; dashed line, limit of detection (500 CFU/ml); asterisks denote lack of detected CFUs. (**b**) Bacterial killing in whole saliva after 2 min treatment. Symbols: white bars, untreated control; gray bars, 0.1 mM, black bars, 1 mM; Con, untreated control; OAK, C_14_KKc_12_K; CHX, chlorhexidine. (**c**) Bactericidal kinetics against *S. mutans* ATCC 35668 upon exposure to chlorhexidine in BHI growth medium. Symbols: squares, untreated control; circles, 20 MIC; triangles, 40 MIC; inverted triangles, 60 MIC; dashed line, limit of detection (500 CFU/ml); asterisks denote lack of detected CFUs. (**d**) Bactericidal kinetics against *S. mutans* ATCC 35668 pre-formed biofilm upon exposure to 0.5 mM of C_14_KKc_12_K or chlorhexidine. Symbols: squares, untreated control; triangles, C_14_KKc_12_K; inverted triangles, chlorhexidine; dashed line, limit of detection (500 CFU/ml); asterisks denote lack of detected CFUs. Results are from at least two independent experiments performed in duplicate. Error bars represent the standard deviation.

Data shown in Fig. 5a confirms the OAK’s ability to maintain a rapid bactericidal activity in a complex medium, such as the supernatant from centrifuged human saliva^52^, albeit at the expense of higher doses compared with BHI medium. Furthermore, using whole saliva instead, the OAK has also maintained the capacity for rapid killing (within 2 minutes) of the multi-organism oral microflora, as efficiently as chlorhexidine (Fig. 5b). Chlorhexidine (CHX) is a cationic polybiguanide used since the 1970’s^53^ as a mouth-wash formulation (1–2 mM) to treat oral inflammations. CHX represents the gold standard reference in the field^54^ despite a few shortcomings such as dental/tissue discoloration and/or negative effects on taste^55, 56^. For these reasons its application is limited to short periods of time (about a week), and is usually employed as part of pre- or post-surgical interventions.

In the literature, CHX MIC against *S. mutans* varies between 0.3 and 4 µM^57^ (0.3 µM, in our hands, representing a nearly 3-fold higher potency than C_14_KKc_12_K). However, when comparing their bactericidal kinetics (Figs 4a and 5c) CHX was bactericidal only at 40 multiples of its MIC (11.2 µM) i.e., at ~10 times the OAK’s bactericidal concentration (1.56 µM). While we are unable to explain this discrepancy, it is possible that these potency manifestations (i.e., the measure of inhibitory concentration versus time-kill kinetics) reflect an aptitude to compensate one for the other (likely related to mechanistic differences).

Moreover, noteworthy is the fact that, when compared at high concentrations (e.g., ≥0.5 mM) C_14_KKc_12_K and CHX were (again) equipotent in their ability to affect viability of *S. mutans* in a preformed biofilm, killing >99% of the initial massive inoculum (10^9^ CFU/ml) within one hour of exposure (Fig. 5d).

## Discussion

Antibacterial membrane-active lipopeptides are currently gaining extensive interest for their potential to affect critical bacterial processes ranging from communication^8, 9^ to antibiotic functions, in mono- and combination-therapy^17^. For example, PMB and daptomycin are two naturally occurring bactericidal cyclic lipopeptides, clinically used against GNB and GPB, respectively, with MIC values ranging between 0.12 and 8 µg/ml (PMB)^58^ or 0.015 and 32 µg/ml (daptomycin)^59^. In both cases, the acyl moiety plays a critical role^45, 60^.

Acyl conjugation was also shown to enhance the antimicrobial properties of various AMPs and synthetic mimics alike^31, 61, 62^. For instance, acylated derivatives of dermaseptin, a broad spectrum amphibian AMP, have drastically influenced its activity spectrum, enabling its conversion to specific activity on either GPB or GNB, depending on the acyl selected^63, 64^.

Other interesting studies reported synthetic ultra-short lipopeptides (3–5 residues) with potent activities against plant-related pathogenic fungi^65^ and bacteria^66^, though at higher doses^67^.

The present study provides evidence for the capacity of equivalent lipopeptides (generated *via* the OAK approach) to yield small molecules susceptible to be useful as simple investigation tools to help clarifying debated mechanistic aspects and potentially useful in biomedical applications. Namely, the findings reported herein, establish C_14_KKc_12_K as the shortest broad-spectrum antibacterial OAK known hitherto. The data also represent a quite remarkable yet ill-understood counter-intuitive outcome regarding the relationships between the hydrophobicity and self-assembly of this particular sequence. As expected from their respective elution time in HPLC using a hydrophobic column, C_14_KKc_12_K possesses an intermediate hydrophobicity value compared with C_12_KKc_12_K and C_16_KKc_12_K (Table 2). However, their tendencies for self-assembly, which presumably also depend on hydrophobicity, did not increase correspondingly, as evidenced by light scattering measurements (Fig. 1). Its sharp premature deviancy from linearity (as evidenced by high intensity) suggests that C_14_KKc_12_K forms drastically different aggregates in terms of three-dimensional organization. In contrast, our findings point to a dramatic increase in antibiotic efficiency since C_14_KKc_12_K achieved not only the lowest MIC value for this analog series (e.g., C_14_KKc_12_K MIC over *S. mutans* is 0.78 *versus* 1.56 µM for C_12_KKc_12_K or C_16_KKc_12_K) but has also upgraded its mode of action from bacteriostatic to bactericidal, including on GNB (Table 2). In this respect, the study expands and reinforces previous findings that illustrated the potential of N-terminal acyl conjugation to the core structure of AMPs^63, 68, 69^ or OAKs as a potent and versatile strategy for optimizing the hydrophobic/cationic balance required for antimicrobial properties.

**Table 2:** Modulating biophysical properties of a core sequence by conjugating an N-terminal acyl.

RX^a^	H^b^ (%)	MIC^c^ (µM)		Effect observed at low concentrations (<3 µM)
		*S.m*.	*E.c*.	
HX	27	>50	>50^40^	Not observed (normal growth)
C_8_X	41	50	>50^40^	Weak perturbations of GNB outer membrane (normal growth)
C_10_X	46	6.25	>50^40^	Weak growth inhibition of some GPB and transient membrane damages including partial depolarization of CM (slight delay in GNB growth)
C_12_X	51	1.56	16^40^	Impairing membrane damages leading to a bacteriostatic mode of action in GPB; OM permeabilization & delayed growth in GNB
C_14_X	54	0.78	3.12	High efficacy in membranes disruption leading to a bactericidal mode of action in both GPB & GNB
C_16_X	62	1.56	25^24^	Trend reversal (reduced potency) due to excess hydrophobicity as self-assembly approaches the critical aggregation concentration

^a^R = N-terminal acyl, X = KKc_12_K;

^b^Hydrophobicity, defined as % acetonitrile eluent in C_18_ HPLC column;

^c^Minimal inhibitory concentration as determined by the microdilution method over *S. mutans* and *E. coli* representing GPB and GNB, respectively.

The data also hint to a relationship between antibacterial potency and bacterial efflux function. Although so far, OAK properties were often rationalized strictly in terms of interactions with bacterial membranes, it is now clear that efflux^70, 71^ represents another decisive factor whose contribution to the mode of action must be accounted for. Previously, we proposed that related but borderline-hydrophobic analogs (e.g., C_12ω7_KKc_12_K^18^ or C_10_KKc_12_K^40^) are substrates of the AcrAB-TolC system, the resistance nodulation division (RND) efflux pump present in *E. coli* and various enteric bacteria. The fact that C_12ω7_KKc_12_K was potently active on isogenic mutant strains (whose efflux-pump components were deleted) supports the view that inactivity on normal GNB stems from the OAK’s rapid extrusion by these pumps^18^. Additional support for this view comes from the fact that bactericidal OAKs are also prone to deeper insertion within the CM^17^. In this respect, the fact that C_14_KKc_12_K is equally potent on both the wild type (AG100) and its deletion-mutant strain (AG100A) reinforces this hypothesis. Combined, our findings hint to a scenario implicating a simultaneous/competitive attraction of the OAK molecules in the periplasm, to the CM anionic phospholipids and the membrane-embedded efflux pumps. Consequently, borderline-hydrophobic OAKs would be more susceptible to extrusion, whereas outright-hydrophobic OAKs (e.g., C_14_KKc_12_K) are more likely to escape extrusion due to their tighter/deeper anchoring within the CM.

Considering the OAK properties in the context of a series of analogs (as outlined in Table 2), the resulting perspective provides an overall rational picture describing a bell-shaped continuum of effects that exacerbate (possibly through accumulation) with increasing hydrophobicity. Thus, the least hydrophobic among the tested lipopeptides (C_8_KKc_12_K) was clearly devoid of growth inhibitory activity against GNB but managed to induce mild damages, mostly to the OM^40^ without affecting bacterial proliferation. While these effects are readily reparable, they may cause some delay in initial bacterial doubling time upon minor hydrophobicity increase (as observed with C_10_KKc_12_K) where they start affecting the CM as well, as expressed by partial depolarization^40^. Additional increase in hydrophobicity (to yield C_12_KKc_12_K) further intensifies these effects to cause a variety of non-reparable damages to both GNB and GPB as expressed by a full-fledged growth inhibitory activity (i.e., MIC). These damages are nonetheless not severe enough to jeopardize bacterial viability, hence often leading to a bacteriostatic mode of action, as observed previously^40^ and in this study. Owing to its optimal hydrophobicity level, C_14_KKc_12_K was able to induce the most severe damages that culminated in rapid bactericidal outcomes. In contrast, excess hydrophobicity (e.g., in C_16_KKc_12_K^24^) leads to the formation of tight peptide aggregates, which in turn, inverse the MAC potency-trend by limiting OAK’s availability for optimal interactions with bacterial targets.

In conclusion, our results support the view that N-terminal acyl-manipulations of the core sequence KKc_12_K, exhibit straightforward structure-activity relationships. Thus, simply by switching the N-terminal acyl, the OAK properties became tunable, gradually evolving from lack of “visible” antibiotic activity on to exerting bacteriostatic activity over GPB only, and ultimately, exercising broad-spectrum bactericidal activity. The fact that C_14_KKc_12_K was equally potent on both wild type and efflux mutant strains suggests that stronger anchoring within the CM enables hydrophobic MACs to escape extrusion by RND pumps, thereby providing a rational for the observed increased potency.

Besides their potential role as investigation tools, such compounds may be useful in treating infections involving multiple microbial populations, such as oral mucositis. Our findings may have relevance to various biofilm-associated micro-environmental niches that hamper drug efficacy in infections or industry related issues. Future studies might clarify this issue. Interestingly, a 35-residue-long MAC currently in phase 2 clinical trials, C16G2^72, 73^ displayed specific antistreptococcal bactericidal properties in saliva. In this respect, the OAK platform might present advantages in the capacity to generate superior anti-biofilm candidates, including in terms of biological robustness, simplicity and production costs.

## Materials and Methods

### Peptide synthesis

OAKs were synthesized in-house (433 A Peptide Synthesizer; Applied Biosystems, Foster City, CA, USA) by the solid-phase method using 9-fluorenylmethyloxycarbonyl (Fmoc) active-ester chemistry on 4-methylbenzhydrylamine (MBHA) resin. OAKs were then deprotected and cleaved from the resin using trifluoroacetic acid:H_2_O (95:5) and purified to >95% chromatographic homogeneity by reverse phase high performance liquid chromatography (RP-HPLC) using C_18_ column (Vydac), a flow rate of 2 ml/min and a linear acetonitrile gradient of 1%/min (Alliance; Waters, Milford, MA, USA). Peaks identity was verified by mass-spectrometry (Xevo G2 Tof; Waters, Milford, MA, USA). Purified OAKs were then lyophilized and kept as dry powder at −20 °C.

### Organization in solution

To assess the OAK’s self-assembly in solution, serial two-fold dilutions of the OAK (initial concentration of 200 µM) were prepared in phosphate buffered saline (PBS; 10 mM Na_2_HPO_4_, 154 mM NaCl, pH = 7.4) and incubated for 2 hr at room temperature (RT). Light scattering at a 90° angle was measured through a 1 nm slit while holding both excitation and emission at 400 nm (Spectrophotometer Fluorolog-3 FL3–22; Horiba Jobin Yvon, Edison, NJ, USA).

To evaluate the disassembly of these aggregates upon bacterial exposure, an OAK solution (200 µM) was incubated (2 hr in PBS, at RT) after which, bacteria were added (10^5^ CFU/ml) and the light scattering evolution of these suspensions was monitored as described above.

### Bacteria

Gram-positive bacteria tested were: American Type Culture Collection (ATCC) strains *Staphylococcus aureus* 25923, 29213, MRSA 39592, 43300, BAA-43 (HSJ 216), BAA-1720 (252), *S. epidermidis* 12228, *S. xylosus* 29971, *Enterococcus faecalis* 29212, *E. faecium* 35667, *Bacillus subtilis* 33677, *B. cereus* 11778, *Listeria grayi* 19120, *L. innocua* 33090, *L ivanovii* 19119, *L. monocytogenes* 19115, *L seeligeri* 35967, *L. welshimeri* 35897, *Streptococcus agalactiae* 13813, 27956, *S. bovis* 9809, ***S. mutans*** **35668, 700610 (UA159)**, *S. pneumoniae* 49619, 6303, *S. pyogenes* 19615, ***S. sobrinus*** **27352 (6715)** and clinical isolates MRSA 10017 (USA300), 15903, VRE Nu28.

Gram-negative bacteria tested were: ATCC strains *Escherichia coli* 25922, 43894, *Pseudomonas aeruginosa* 27853, 9027, *Acinetobacter baumannii* 19606, *A. caloaceticus* 31299, *Salmonella* Typhimurium 14028, ***Fusobacterium nucleatum*** **23726**, ***Porphyromonas gingivalis 53977***, clinical isolates *E. coli* 14182, 14384, U-16327, U-16329, *P. aeruginosa* 1278, *Klebsiella pneumoniae* 1287, K2–224, C2, *A. baumannii* 1279, 1280, 1281, ***F. nucleatum*** **PK 1594**, the engineered *E. coli* ML-35p and the isogenic K-12 pair AG100, AG100a (ΔacrAB). Dental related bacteria (generous gift of Prof. Doron Steinberg and Gilad Bachrach from the Hebrew University of Jerusalem) appear in bold characters.

### Culture conditions

*Staphylococci*, *Bacilli*, *Escherichia*, *Pseudomonas*, *Acinetobacter*, *Salmonella* and *Klebsiella* species were grown in Luria Bertani broth (LB; 5 gr/l NaCl, 5 gr/l yeast extract, 10 gr/l tryptone). *Enterococci* and *E. coli* ML-35p were grown in Tryptic Soy Broth (TSB). *Listeria* and *Streptococci* were grown in Brain Heart Infusion (BHI). All bacteria were grown over-night at 37 °C with shaking. *S. mutans* 35668 plated on BHI agar for enumeration was grown for 48 hr. *S. mutans* UA159 was grown in 5% CO_2_ enriched atmosphere. *Fusobacterium* and *Porphyromonas* were grown in Wilkins-Chalgren growth medium and an anaerobic atmosphere.

**Minimal inhibitory concentration** (MIC) was determined using the microdilution assay. Mid-log-phase bacteria at 5 × 10^5^ CFU/ml were incubated in a 96-well plate with serial two-fold dilutions of the tested compound for 18–24 hr at 37 °C (final volume of 200 µl). O.D. at 620 nm was measured (Synergy HT, BioTek Instruments, Winooski, VT, USA), and the MIC was determined as the lowest concentration for which no increase in O.D. was detected.

**Bactericidal kinetics** was assessed by incubating 5 × 10^5^ CFU/ml of mid-log-phase bacteria with the OAK for 3 hr at 37 °C with shaking. Aliquots were taken at t = 0, 0.5, 1, 2 and 3 hr, subjected to serial 10-fold dilutions in saline (NaCl = 0.85%) and plated for enumeration after 24–48 hr incubation at 37 °C.

**Cytoplasmic membrane permeation to ethidium bromide** (EtBr) was evaluated as follows: Mid-log-phase bacteria at 1 × 10^8^ CFU/ml were centrifuged for 5 min at 15,000 g. Pellet was washed twice with PBS containing 0.5% glucose (pH = 7.4), suspended in the same buffer and incubated for 10 min at 37 °C with shaking. 180 µl of the bacterial suspension were mixed in a 96-well plate with 25 µl of the tested compound and EtBr (EtBr final concentration = 1 µg/ml) and fluorescence was recorded immediately (excitation: 535 nm, emission: 590 nm) for up to 30 min at 37 °C with shaking (Synergy HT, BioTek Instruments, Winooski, VT, USA).

**Outer and cytoplasmic membrane permeation in Gram-negative bacteria** was assessed using the engineered *E. coli* ML-35p by monitoring the chromogenic hydrolysis of two indicators: ortho-nitrophenyl-β-galactoside (ONPG) and nitrocefin. Mid-log-phase *E. coli* ML-35p were centrifuged for 5 min at 15,000 g and the supernatant was removed. Pellet was washed three times with sodium phosphate buffer (SPB; 10 mM NaH_2_PO_4_, pH = 7) and suspended in the same buffer (O.D. at 620 nm = 1). Bacteria were then 10-fold diluted into SPB containing 3% TSB. 100 µl of bacterial suspension were placed in a 96-well plate with 100 µl of the tested compound and 20 µl of either ONPG (final concentration = 2.5 µM) or nitrocefin (final concentration = 25 µM). Hydrolysis of ONPG and nitrocefin was monitored immediately by recording the absorbance at 420 nm and 486 nm respectively, for 30 min at 37 °C with shaking (Synergy HT, BioTek Instruments, Winooski, VT, USA).

**Cytoplasmic membrane depolarization** was assessed by monitoring the displacement of the membrane binding fluorescent dye DiSC_3_(5) (3,3′-dipropylthiadicarbocyanine iodide) as follows. Mid-log-phase bacteria at 5 × 10^8^ CFU/ml were centrifuged for 5 min at 15,000 g. Pellet was washed twice with 5 mM 4-(2-hydroxyethyl)piperazine-1-ethanesulfonic acid (HEPES) containing 5 mM glucose (pH = 7.2) and suspended in the same buffer. Bacteria were diluted 10-fold in the same buffer, DiSC_3_(5) was added to a final concentration of 4 µM and samples were incubated at RT in the dark for 1 hr. KCl was added to a final concentration of 100 µM and incubation continued for an additional hour. 180 µl of bacterial suspension were placed in a 96-well plate and fluorescence was recorded until signal stabilization (excitation: 620 nm, emission: 680 nm). Then, 20 µl of the tested compound were mixed into the wells and fluorescence was recorded immediately for up to 30 min at 37 °C with shaking (Synergy HT, BioTek Instruments, Winooski, VT, USA).

**Dansyl-polymyxin displacement assay** was assessed by displacement of dansyl-polymyxin B bound to lipopolysaccharide (LPS) as follows. Polymyxin B sulfate was covalently attached to dansyl chloride and mono-dansyl Polymyxin B (DPMB) was purified by RP-HPLC. 180 µl of 5 mM HEPES containing 3 µg/ml LPS (from *E. coli* or *P. aeruginosa*) and 2 µM mono-DPMB were incubated in a 96-well plate with 20 µl of the tested compound for 1.5 hr at RT and fluorescence (excitation: 340 nm, emission: 485 nm) was measured immediately (Synergy HT, BioTek Instruments, Winooski, VT, USA).

**Bactericidal kinetics in saliva** was assessed as follows. Whole saliva was pooled from two-three healthy volunteers at a time, after obtaining their informed consent. To test for specific activity against *S. mutans*, saliva was centrifuged for 5 min at 15,000 g and 4 °C, and the supernatant was spiked with 1 × 10^7^ CFU/ml of mid-log-phase *S. mutans* 35668. Otherwise, activity against natural oral microflora was also performed on whole saliva. Saliva was mixed with the tested compound, incubated at 37 °C with shaking, and aliquots were taken at t = 0, 2, 5 and 10 min. Aliquots were subjected to serial 10-fold dilutions in saline and plated on BHI agar for enumeration.

**Anti-biofilm activity** was evaluated against established biofilms in 96-well plates as follows. 200 µl of mid-log-phase *S. mutans* 35668 at 5 × 10^5^ CFU/ml in BHI containing 2% sucrose were placed in each well, and plates were incubated for 24 hr at 37 °C without shaking. Unattached cells were removed by decanting the plates and biofilms were washed three times with milliQ water. Solutions of the tested compounds were placed on the biofilms for up to 3 hr at 37 °C without shaking. Plates were decanted in 0.5 hr intervals and biofilms were washed three times with milliQ water to remove any compound residues. Biofilms were scraped from the bottom of the wells, suspended in BHI and sonicated for 5 min in a sonication bath, subjected to serial 10-fold dilutions in saline and plated on BHI agar for enumeration.
